# Designing a Recombinant Vaccine against *Providencia rettgeri* Using Immunoinformatics Approach

**DOI:** 10.3390/vaccines10020189

**Published:** 2022-01-25

**Authors:** Saba Gul, Sajjad Ahmad, Asad Ullah, Saba Ismail, Muhammad Khurram, Muhammad Tahir ul Qamar, Abdulrahim R. Hakami, Ali G. Alkhathami, Faris Alrumaihi, Khaled S. Allemailem

**Affiliations:** 1 Department of Health and Biological Sciences, Abasyn University, Peshawar 25000, Pakistan; sabagul0332@gmail.com (S.G.); asadullahaup@gmail.com (A.U.); 2 Department of Biological Sciences, National University of Medical Sciences, Rawalpindi 46000, Pakistan; sabaismail7@gmail.com; 3 Department of Pharmacy, Abasyn University, Peshawar 25000, Pakistan; muhammad.khurram@abasyn.edu.pk; 4 College of Life Science and Technology, Guangxi University, Nanning 530004, China; m.tahirulqamar@hotmail.com; 5 Department of Clinical Laboratory Sciences, College of Applied Medical Sciences, King Khalid University, Abha 61481, Saudi Arabia; ahakami@kku.edu.sa (A.R.H.); agaithan@kku.edu.sa (A.G.A.); 6 Department of Medical Laboratories, College of Applied Medical Sciences, Qassim University, Buraydah 51452, Saudi Arabia; f_alrumaihi@qu.edu.sa

**Keywords:** antibiotic resistance, *Providencia rettgeri*, immunoinformatics, multi-epitope vaccine

## Abstract

Antibiotic resistance (AR) is the resistance mechanism pattern in bacteria that evolves over some time, thus protecting the bacteria against antibiotics. AR is due to bacterial evolution to make itself fit to changing environmental conditions in a quest for survival of the fittest. AR has emerged due to the misuse and overuse of antimicrobial drugs, and few antibiotics are now left to deal with these superbug infections. To combat AR, vaccination is an effective method, used either therapeutically or prophylactically. In the current study, an in silico approach was applied for the design of multi-epitope-based vaccines against *Providencia rettgeri*, a major cause of traveler’s diarrhea. A total of six proteins: fimbrial protein, flagellar hook protein (FlgE), flagellar basal body L-ring protein (FlgH), flagellar hook-basal body complex protein (FliE), flagellar basal body P-ring formation protein (FlgA), and Gram-negative pili assembly chaperone domain proteins, were considered as vaccine targets and were utilized for B- and T-cell epitope prediction. The predicted epitopes were assessed for allergenicity, antigenicity, virulence, toxicity, and solubility. Moreover, filtered epitopes were utilized in multi-epitope vaccine construction. The predicted epitopes were joined with each other through specific GPGPG linkers and were joined with cholera toxin B subunit adjuvant via another EAAAK linker in order to enhance the efficacy of the designed vaccine. Docking studies of the designed vaccine construct were performed with MHC-I (PDB ID: 1I1Y), MHC-II (1KG0), and TLR-4 (4G8A). Findings of the docking study were validated through molecular dynamic simulations, which confirmed that the designed vaccine showed strong interactions with the immune receptors, and that the epitopes were exposed to the host immune system for proper recognition and processing. Additionally, binding free energies were estimated, which highlighted both electrostatic energy and van der Waals forces to make the complexes stable. Briefly, findings of the current study are promising and may help experimental vaccinologists to formulate a novel multi-epitope vaccine against *P. rettgeri*.

## 1. Introduction

Antibiotic resistance (AR) is the defense mechanism pattern in microbes, and it occurs when bacteria, fungi, or viruses evolve over some time, such that it protects the microorganism against antibiotics adapts itself to the environmental conditions [1]. Antibiotics are medicines used to treat bacterial infections. All commercially available antibiotics are becoming ineffective, as multi-drug resistant strains of microbes are spreading worldwide, leading to bacterial and fungal diseases with less-effective treatments [2]. This phenomenon has mainly sped up due to misuse and overuse of antibiotics [3]. AR is an alarming global challenge associated with a high mortality and morbidity rate in humans and animals [4]. Antimicrobial resistance (AMR) is becoming difficult to treat with currently available antibiotics due to the high level of genetic diversity in microbial species [5]. The excessive use of antibiotics in humans and animal medicines, agriculture, and the environment has led to AR in bacteria, which has significantly contributed to high hospital and community mortality and mobility [6]. AR is the outcome of the bacterial evolution process, making itself fit to changing environmental milieu in the quest for survival of the fittest [7]. The AR is increasing and together with poor infection-controlled clinical practices, the resistant genetic determinants are spreading fast to non-AR microbes as well as to the environment [8]. Therefore, there is a dire need to devise new strategies for effective management of AR pathogens [3,9,10,11].

Vaccines are a biological preparation that help in generating acquired immunity against any given pathogen or pathological condition [12]. Vaccines consist of either the whole microorganism or a byproduct of the microbes, e.g., toxins, etc. [13]. A vaccine is typically made from the inactivated, killed, or weakened toxins or surface proteins of the infectious agent that have only the ability to provoke the immune response of the host [14]. A potential vaccine candidate taken for the preparation of an efficient vaccine must fulfill the criteria of certain parameters, e.g., the vaccine candidates should be highly antigenic, conserved, and non-homologous to the host proteome and its normal flora (which are essential for microbe survival) and should it be easily recognizable by the immune cells of the host [15]. An effective and safe vaccine discovery against poliovirus by Salk and Sabin was carried out by Pasteur’s vaccinology concept [16]. Pasteur’s rule of vaccines led to the development of the BCG vaccine against *Mycobacterium tuberculosis* [17], along with the vaccine against mumps, measles, and rubella [18]. However, such vaccinology is a failure for pathogens that are unable to be cultured or grown in the laboratory [19]. In addition, culture-based developed vaccines exhibit antigenic variability [20]. Particularly, Pasteur vaccinology limitations have surfaced in the cases of *Neisseria meningitides* and *Mycobacterium leprae*. Subunit vaccines that target cellular components contain virulence factors [21], as can be exemplified by the pertussis vaccine [22], outer membrane Meningococcal Vesicle (OMV)-containing protein, PorA/porin, and pneumococcal polysaccharide vaccine (PPSV23) [23]. A potential vaccine candidate taken for the preparation of an efficient vaccine must fulfill the criteria of certain parameters, e.g., the vaccine candidates should be highly antigenic, conserved, and non-homologous to the host normal flora and proteome (which are essential for microbe survival) and should be easily recognizable by the immune cells of the host [15]. 

To combat AR and to eliminate this alarming global health issue, both therapeutically and prophylactically, the introduction of novel approaches remains vital [24]. Boosting host immunity via immunotherapeutic and immunological interventions among various approaches proposed by the National Institute of Allergy and Infectious Diseases (NIAID) is an attractive solution to fight the challenges of AR [25]. Reverse vaccinology (RV) is an up-to-date approach and is used to identify putative surface-associated proteins without the need to culture the microorganisms [26]. Through immunotherapy, pathogen-specific antibodies can be designed and produced; meanwhile, immunological interventions in the human immune system are trained to tackle bacterial infections by acquiring adaptive immunity [27]. The latter strategy, using vaccines in particular, holds great importance in lowering the burden of AR [28]. Conventional vaccine development is expensive, time consuming, and needs many human resources [21]. The genomic revolution will greatly aid in disclosing new vaccine candidates that, by traditional vaccine development, are hard to detect [29]. Next-generation sequencing of bacterial pathogens and advanced bioinformatics practices in vaccinology are now commonly employed for the identification of putative surface-associated antigens [30]. The meningococcal serogroup B (4CMenB) vaccine was effectively developed using the RV approach [31]. Pan-genomic reverse vaccinology (PGRV), specifically, is more effective compared to conventional RV, as it screens highly conserved targets that are strain specific [32]. For example, the genome of *Streptococcus agalactiae* revealed four protective antigens identified via the PGRV approach [6].

Providencia is a group of opportunistic urease-producing Gram-negative, motile, and rod-shaped bacteria belonging to the *Enterobacteriaceae* family. *P. rettgeri* can cause a variety of hospital-acquired infections, i.e., urinary tract infections, wounds, human blood infections, gastroenteritis, and bacteremia [33] *P. rettgeri* is ubiquitous in the environment. It may exist in both land and water habitats and may therefore be present in water, soil, and land, most necessarily in hospital and nursing vicinities [34]. *Providencia* shows resistance to several antibiotics and can cause several types of hospital-associated infections with high mortality and morbidity rates [35]. *P. rettgeri* is resistant to commercially available antibiotics such as ampicillin, polymyxins, first-generation cephalosporins [36], and gentamicin, along with tobramycin, ciprofloxacin, levofloxacin [37], carbapenem, polymyxins, and tigecycline [38]. Additionally, it is resistant to amikacin [39]. Compared to other bacterial species such as *Klebsiella pneumonia* and *Acinetobacter baumannii*, *P. rettgeri* is naturally resistant to tigecycline and colistin [39]. According to previous literature, about 86% of the isolates of *P. rettgeri* were found to be resistant to amikacin antibiotics; in addition, 71% of the clinical isolates were resistant to gentamycin drugs [34]. Furthermore, metallo-β-lactamase-1 was noted as NDM-1. Extended-spectrum beta-lactamases and amoxicillin-clavulanate resistivity patterns have also been reported [40]. The outcome of this study will provide a ready-to-use multi-epitope peptide vaccine for experimentalists to evaluate its real immune protection ability in animal models. This will not only enrich the vaccine antigens against *P. rettgeri* but will also speed up the vaccine development process without consuming much cost and technical expertise. From a clinical usefulness perspective, the vaccine will likely provide protection against all sequenced strains of the pathogen, as it is based on the core genome. The vaccine is composed of safe antigens and will be less risky compared to other types of vaccine. Furthermore, such vaccines are easy to produce and can be inserted into multiple carrier systems. These findings will also lead in the development of economically cheaper vaccines, will aid in stopping the spread of AR bacterial strains and will thus be useful in saving millions of lives.

## 2. Research Methodology

**Figure 1:**
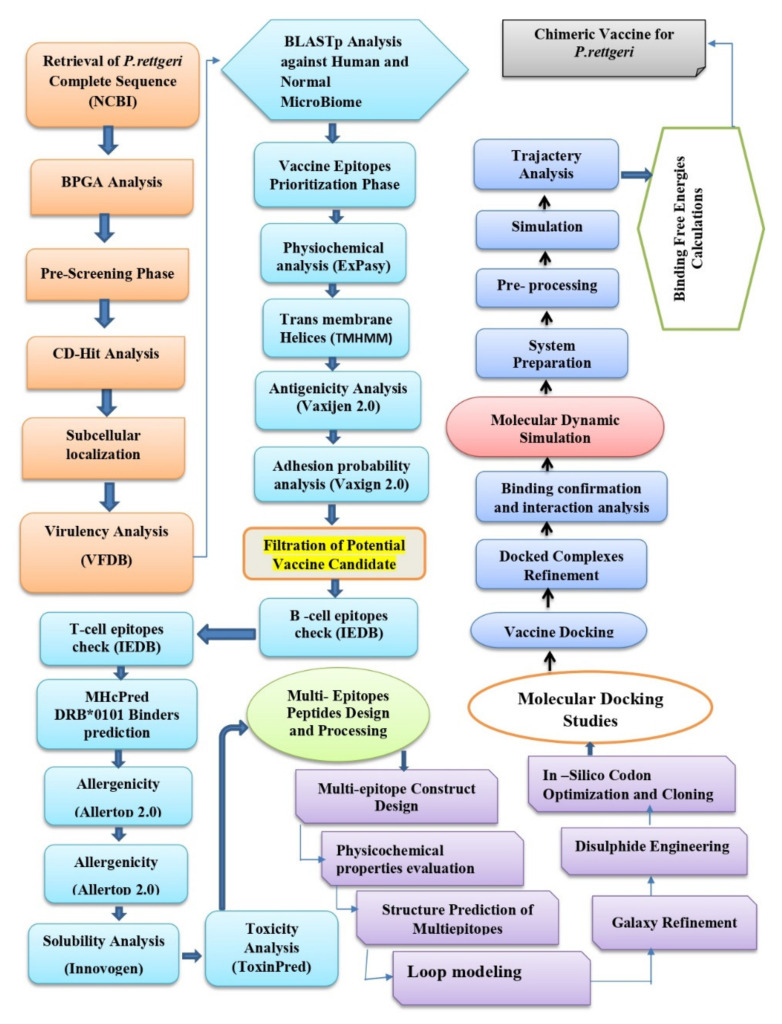
Overall flow of methodology that was used in the design of the multi-epitope vaccine against *P. rettgeri*.

The methodology flow that is used in the design of the multi-epitope vaccine against *P. rettgeri* is mentioned in Figure 1.

### 2.1. P. rettgeri Complete Proteome Retrieval

This research commenced with the retrieval of complete proteomes of *P. rettgeri*. The bacteria have a total of 14 strains, and their genomic/proteomic data were retrieved from the genome database of the National Center For Biotechnology Information (NCBI) database [41]. All the respective proteomes and genomes were retrieved in FASTA format [42].

### 2.2. BPGA Analysis 

Bacterial pan-genome analysis is a bioinformatics approach mainly used for the retrieval of core, unique and accessory genes. Herein, the bacterial pan-genome analysis was considered to investigate the pan-genome of the pathogen [19]. Through the one-click analysis technique of the BPGA tool [43], all 14 strains of *P. rettgeri* were analyzed for core genome (conserved), along with accessory genes (dispensable) and unique genes (strain-specific genes) [44].

### 2.3. Pre-Screening Phase 

The pre-screening phase was considered as the filtering phase/subtractive proteomic phase in which several potential antigenic targets were determined in the core genome of the pathogen and were prioritized as the potential vaccine candidates. This phase consisted of CD-HIT analysis [45], homology check, essential check, and surface localization check [23]. 

### 2.4. CD-Hit Analysis (Cluster Data with High Identity and Tolerance)

The bacterial core genome mostly consists of redundant and non-redundant proteins. Redundant proteins have double representation in the core proteome, which is not important for further processing in the design of the vaccine candidate. To remove all the redundant proteins, the CD-Hit-h analysis approach was utilized. Furthermore, the non-redundant proteins were considered for further processing [46]. 

### 2.5. Subcellular Localization Analysis

Surface-localized proteins can be easily recognized by the host immune system, and these surface-localized proteins are mainly involved in the pathogenicity of infection. Hence, due to being pathogenic in nature and surface localization, these proteins were prioritized as vaccine candidates. This task was achieved through PSORTb analysis [47].

### 2.6. Virulent Protein Analysis 

The virulent factor database (VFDB) is a dataset for bacterial and fungal virulence factors. In virulent factor analysis, the prioritized subcellular localized proteins were examined for virulence through Basic Local Alignment Search (BLASTp) against the VFDB full proteomic dataset [48]. The cut-off values kept for prioritization were: bit score ≥ 100 and sequence identity ≥30 percent. The proteins that failed to fulfill the noted criteria were discarded [49]

### 2.7. BLASTp Analysis against Humans and MicroBiome

The BLASTp tool was used to check the homology of filtered virulent proteins against human normal flora and the proteome. The homology check was performed to prevent the chances of autoimmune responses by the host against self-antigens. In this analysis, similar to the selected proteins, it was checked with humans and three normal intestinal flora; *Lactobacillus rhamnosus* (taxid: 47715), *L. johnsonii* (taxid: 33959), and *L. casei* (taxid: 1582) [50]. The cut-off sequence identity and E values used were at ≤30% and 10^−4^, respectively.

### 2.8. Vaccine Epitopes Prioritization Phase

Vaccine candidate prioritization is the proceeding step for designing a multi-epitope vaccine. In this phase, the shortlisted proteins were further subjected to physicochemical analysis, transmembrane helices analysis, antigenicity, allergenicity, adhesion probability, and B-cell and T-cell epitopes prediction [51].

### 2.9. Physiochemical Analysis

Physiochemical analysis of the shortlisted proteins was performed for properties such as instability index, atomic composition, amino acid composition, theoretical PI, molecular weight, aliphatic index, estimated half-life, and grand average of hydropath city (GRAVY). This task was achieved with the help of an online web server, ProtParam [52]. The protein was identified as a good vaccine target when the cut-off value of the protein molecular weight was <110 kDa and when the instability index was <40 [53]. All the unstable proteins were excluded from the study, and stable proteins were subjected to further analysis.

### 2.10. Transmembrane Helices

HMMTOP 2.0 and TMHMM 2.0 tools were used to check for transmembrane helices [54]. The cut-off values for the number of transmembrane helices were 0 or 1, and proteins exceeding these values were discarded. The proteins were regarded as good vaccine candidates only when the transmembrane helices were less than the threshold, as such proteins can be easily handled in experimental studies. The selected proteins were further processed in downward analysis [55].

### 2.11. Antigenicity Prediction

Antigenicity is the capability of foreign antigens to bind immune cells and generate proper immune responses. Antigenicity prediction was performed through an online Vaxijen 2.0 webserver (http://www.ddg-pharmfac.net/vaxijen/VaxiJen/VaxiJen.html, accessed on 18 June 2021) [16]. To prioritize potential vaccine candidates, ≥0.4 cut-off value was considered [56]. The higher the antigenicity of the vaccine construct is, the stronger the chances of host–immune system provocation [26]. 

### 2.12. Adhesion Probability Analysis

The adhesion probability was the next step followed for selecting and prioritizing proteins that had the ability of adhesion. The threshold value to prioritize good adhesive candidates was >0.5 [57]. This ensures active attachment of the designed vaccine with the host immune cells and ensures robust host immunity. The adhesion and binding with the host immune system developed adaptive immunity, inducing both antibodies and TCR (T-cell receptors). This task was achieved using an online Vaxign webserver [58].

### 2.13. Epitopes Prediction Phase 

In the epitopes prediction phase, both B- and T-cell epitopes were predicted through the online immune–epitope database (IEDB) tool [59]. The IEDB covers a wide-ranging collection of immune epitopes and can be easily accessed. First, B-cell epitopes were predicted and B-cell epitopes were further used for T-cell epitope prediction [60]. The predicted epitopes were prioritized based on low percentile rank. Furthermore, through the MHcPred tool, binding potency of the predicted epitopes with DRB*10101 alleles was analyzed [61,62]. Finally, in epitope prediction, shortlisted epitopes were subjected again to antigenicity, virulence, toxicity, and water solubility analysis through Vaxijen 2.0, Virulentpred, ToxinPred, and ProteinSol, respectively [63].

### 2.14. Multi-Epitope Peptide Designing and Processing

Multi-epitope-based vaccines consist of several antigenic epitopes [64]. In the multi-epitope design phase, all the screened epitopes were linked via GPGPG linkers to construct a multi-epitope-based vaccine. The designed vaccine construct was further linked to cholera toxin B subunit adjuvant to enhance immunogenicity of the vaccine. 

### 2.15. Physiochemical Properties of the Final Vaccine Construct

The physiochemical properties such as number of amino acids, molecular weight, theoretical PI value, instability index, aliphatic index, and grand average of hydropathicity (GRAVY) were analyzed [53]. This task was achieved through an online webserver, ProtParam [65]. 

### 2.16. Structure Modeling of the Final Vaccine Construct

The 3D structure of the vaccine construct was modeled using the 3Dpro tool to predict the most stable vaccine structure, as it is essential for molecular recognition. The 3D structure was checked to ensure the construct’s stability and durability [19].

### 2.17. Loops Modeling

The vaccine was passed through Galaxy Loop of the GalaxyWeb server to remove the unnecessary loops from the vaccine construct [66]. This was vital to obtain the most suitable vaccine 3D structure. 

### 2.18. Galaxy Refinement

The final loop-modeled vaccine construct was further analyzed in GalaxyRefine of GalaxyWeb server. The vaccine was reconstructed for its side chains, and steric clashes were removed. The refined vaccine construct was considered as a good and potential vaccine candidate [67].

### 2.19. Disulfide Engineering

To enhance stability of the vaccine, disulfide engineering was performed. The stability of the vaccine was enhanced by reducing the conformational energy of the twisted and folded structures. For enhanced and improved stability, both inner and outer chain bonding was examined computationally. The Design 2.0 webserver was used for disulfide engineering [68]. 

### 2.20. Codon Optimization

The Java Codon Adaptation Tool (JCat) server was used to convert the multi-epitope vaccine sequence into DNA and was then cloned in the *Escherichia coli* expression system. The vaccine maximum expression is in the *E. coli* expression system and was calculated with the codon adaptation index (CAI) and its GC percentage value. The value of 1 was estimated as an ideal value for the said expression system [69].

### 2.21. Molecular Docking

Molecular docking studies were applied in order to check the binding efficacy of the designed vaccine construct with different types of immune cell receptors. A blind docking approach was used to predict the binding affinity of the vaccine to TLR-4 (4G8A), MHC-I (1I1Y), and MHC-II (1KG0) receptors retrieved from PDB. The docking study was performed through an online web server of PatchDock [70]. The docking works on the following principles: sample conformation and function scoring. The docked complexes were then refined via the FireDock server [71]. 

### 2.22. Molecular Dynamic Simulation

Molecular dynamics simulation (MDS) is a computational approach to predict the movement of molecules and atoms. In MDS, the molecules and atoms interact for a specific period. MDS of the vaccine with innate immune receptors was carried out using AMBER 20 [72]. The FF14SB was used as a force field, while TIP3P water box (12 Angstrom is size) was used for vaccine–receptor complex submersion. The SHAKE algorithm was used for constraining hydrogen bonds. The complexes were heated and equilibrated, and then a production run was performed for 250 ns. CCPTRAJ was used for trajectory analysis. 

### 2.23. Binding Free Energies Calculation

Additionally, the intermolecular affinity of the vaccine–receptor complex was validated through the AMBER MMPBSA.py module [73]. The binding free energies demonstrate and ensure the system stability, and the lesser the energy is, the more stable the vaccine construct. [74]. In total, 100 frames were analyzed during this analysis. 

### 2.24. C-Immune Simulation

C-ImmSim server was used to decipher host–immune responses to the designed vaccine [75]. This server was used for antigen characterization and its profiling to calculate immune responses of a host, i.e., human. The server was used to run the process of simulation for three separate mammalian organs, i.e., lymph nodes, thymus, and bone marrow, while the parameters used for the process of the simulation were by default [76].

## 3. Results

AR in bacterial species is becoming a worldwide public health concern [77]. One example is the rapid emergence of AR in *P. rettgeri*, resulting in pathogenic hospital-associated infections [78]. As no licensed vaccine is available against the pathogen, computational vaccine design is an alternative approach to speed up vaccine development [79]. In the current research study, we have designed a computational-based multi-epitope recombinant vaccine against *P. rettgeri*. 

### 3.1. Providencia rettgeri Complete Proteome Retrieval

In the current research, the complete proteome of 14 strains of *P. rettgeri* was retrieved from the NCBI databases (https://www.ncbi.nlm.nih.gov/) (accessed on 4 April 2021), followed by a pan-genome analysis.

### 3.2. Bacterial Pan Genome Analysis

The total proteome size of *P. rettgeri* is 13,580 proteins, as mentioned in Figure S1, while all 14 bacterial strains and their genome size are figuratively represented and presented in Figure 1. Due to genome plasticity, there are high chances of gaining new genes over time because the pathogen genomes are open, which is indicated in the core-pan plot. Moreover, the core proteins are typically involved in metabolic regulation in addition to metabolic biogenesis, which has been tested by the COG distribution analysis [80]. The storage and processing of information genes are present, mainly in unique proteins set. The processing of RNA and the process of replication, transcription and translation of recombination genes are all part of the core genome. Likewise, the pan-phylogeny tree of all 14 *P. rettgeri* is shown in Figure S1B.

### 3.3. CD-HIT Analysis

**Figure 2:**
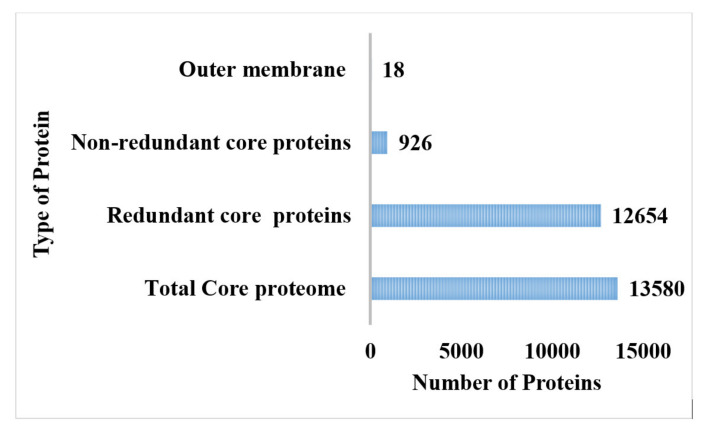
Number of the total proteome, core proteome, and redundant, non-redundant, and virulent proteins.

The CD-hit analysis was accomplished for the retrieval of core proteomes without duplicate sequences [81]. The core proteome of *P. rettgeri* comprised 926 non-redundant proteins, while 12,654 were found to be redundant proteins, as shown in Figure 2. The redundant protein sequences were discarded from the study because they were not required in the vaccine development process due to repeating copies of the same proteins, while non-redundant core proteins were further used in the subcellular localization phase and virulent analysis [82].

### 3.4. Subcellular Localization

The surface or membrane proteins are easily recognized by the host immune system; hence, potential immune responses are generated when these proteins are used in the design of a vaccine. Subcellular localization analysis was performed as an essential check for surface proteins [83]. In the non-redundant core proteome, seven were found to be extracellular, 18 were in the outer membrane, and 25 periplasmic membrane proteins were identified in the subcellular localization analysis.

### 3.5. VFDB Analysis 

Virulent proteins are mainly involved in the pathogenesis of infectious pathogens [48]. In VFDB analysis, these protein sequences were considered as virulent, which fulfilled the bit score >100 percent with the sequence identity of higher than 30% [23]. Among the 50 subcellular localized proteins, only 13 protein sequences were found as virulent.

### 3.6. Human and Normal Flora, Adhesion Probability, Physiochemical Property Analysis 

**Figure 3:**
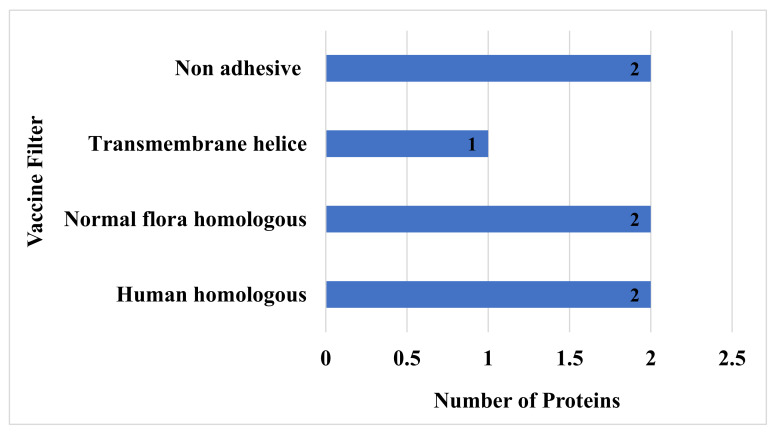
Number of non-adhesive normal flora and homologous proteins that have more than one transmembrane helix.

To avoid the autoimmune response, all the virulent protein sequences were further checked for homology analysis against human and normal flora proteomes [84], and here in this study, only two proteins have shown homology with humans, and two protein sequences have shown similarities with normal flora of host and were hence discarded. The remaining non-similar proteins were subjected to transmembrane helices check, which is essential for feasibility in experimental evaluation [85]. All the sequences that had more than one transmembrane helix were removed, while those that had 0 or 1 transmembrane helices were considered for further analysis [86]. In this step, only one sequence was discarded due to not fulfilling the above criteria. Proteins with more than one transmembrane that showed similarity to humans and their microbiota and that were non-adhesive were discarded. Furthermore, the proteins were predicted to be stable when their instability index was less than 40 with a molecular weight of less than 100 kDa. Physiochemical properties of the filtered protein sequences are mentioned in Table 1. Finally, among the eight filtered proteins, only two proteins were discarded in the adhesion probability check. Concluding, among the total 13 virulent sequences, seven were discarded in the homology check with the host and normal flora proteome, the transmembrane helices check, and the adhesion probability check, as shown in Figure 3.

### 3.7. Vaccine Epitopes Prioritization Phase

In the current study, all six prioritized proteins, which were filtered from the above steps, and checks were subjected to the epitope prioritization phase. In the epitope prioritization phase, both B- and T-cell epitopes were predicted to generate B- and T-cell immune responses [87].

### 3.8. B-Cell Epitopes Prediction

An immune response that is dependent on antibodies may also be referred to as humoral immunity; after stimulation, B-cells convert into plasma cells [88]. From six shortlisted proteins: fimbrial protein, flagellar hook protein (FlgE), flagellar basal body L-ring protein (FlgH), flagellar hook-basal body complex protein (FliE), flagellar basal body P-ring formation protein (FlgA) and Gram-negative pili assembly chaperone domain protein, B-cell epitopes were predicted, as tabulated in Table S1.

### 3.9. T-Cell Epitopes Prediction

The function of the T-cell epitopes is mainly to generate a cellular immune response. T-cell-dependent immunity is also known as cellular immunity. The resultant multiplication and differentiation of T-cell lymphocytes as an outcome of recognizing peptide antigens is to develop the primary immune response [89]. B-cell-derived T-cell epitopes that have the ability to activate the cellular immune response were predicted using B-cell epitopes generated from T-cell epitopes, and therefore MHC-I and MHC-II epitopes were recognized on the basis of lowest percentile scores [90]. The following MHC-I subset alleles are: HLA-A*01:01, HLA-A*01:01, HLA-A*02:01, HLA-A*02:01, HLA-A*02:03, LA-A*02:03, HLA-A*02:06, HLA-A*02:06, HLA-A*03:01, HLA-A*03:01, HLA-A*11:01, HLA-A*11:01, HLA-A*23:01, HLA-A*23:01, HLA-A*24:02, HLA-A*24:02, HLA-A*26:01, HLA-A*26:01, HLA-A*30:01, HLA-A*30:01, HLA-A*30:02, HLA-A*30:02, HLA-A*31:01, HLA-A*31:01, HLA-A*32:01, HLA-A*32:01, HLA-A*33:01, HLA-A*33:01, HLA-A*68:01, HLA-A*68:01, HLA-A*68:02, HLA-A*68:02, HLA-B*07:02, HLA-B*07:02, HLA-B*08:01, HLA-B*08:01, HLA-B*15:01, HLA-B*15:01, HLA-B*35:01, HLA-B*35:01, HLA-B*40:01, HLA-B*40:01, HLA-B*44:02, HLA-B*44:02, HLA-B*44:03, HLA-B*44:03, HLA-B*51:01, HLA-B*51:01, HLA-B*53:01, HLA-B*53:01, HLA-B*57:01, HLA-B*57:01, HLA-B*58:01, HLA-B*58:01; and MHC-II alleles are: HLA-DRB1*01:01, HLA-DRB1*03: *04:01, HLA-DRB101,HLA-DRB1*04:05, HLA-DRB1*07:01, HLA-DQA1*03:01/DQB1*03:02, HLADQA1*03:01/DQB1*03:02, HLADQA1*01:02/DQB1*06:02, HLADPA1*02:01/DPB1*01:01, HLADPA1*01:03/DPB1*04:01, HLADPA1*03:01/DPB1*04:02, HLADPA1*02:01/DPB1*05:01, HLADPA1*02:01/DPB1*14:01. MHC-I and MHC-II molecules of the T-cell are tabulated in following Table S2. 

### 3.10. Epitope Prioritization Phase

In the epitope analysis and prioritization phase, all the predicted B- and T-cell shortlisted epitopes were next subjected to further analyses, such as DRB*0101 binding affinity [91] followed by allergenicity, solubility, and toxicity analyses [92].

### 3.11. DRB*0101 Binding Analysis

Vaccine binding affinity to host immune cell receptors is important for a proper immune response. In the DRB*0101 analysis, all selected epitopes were further checked for the potential of binding with the HLA DRB*0101 allele [93,94]. Only those epitopes of IC50 values <100 nM for DRB*0101 alleles were selected, as they represent strong binding [95]. The shortlisted epitopes whose values are less than the above-mentioned threshold are listed in Table 2.

### 3.12. Antigenicity, Allergenicity, Solubility, and Toxicity Analysis of Selected Epitopes

Only antigenic proteins can stimulate host immune responses [73]. To achieve this task, only antigenic proteins were included in the study, and all probable non-antigenic protein sequences were excluded. To avoid allergic and toxic responses, allergenicity and toxicity analyses were performed for removal of all toxic and allergic protein sequences, as well as poor water soluble epitopes [92,96]. To achieve solubility prediction, an online webserver InvivoGen was used, which is available at https://www.invivogen.com/ova-peptide and accessed on 7 June 2021. All shortlisted probable antigenic, non-allergic, nontoxic, and good water-soluble peptides are mentioned in Table 2.

### 3.13. Multi-Epitope Vaccine Construction and Processing

**Figure 4:**
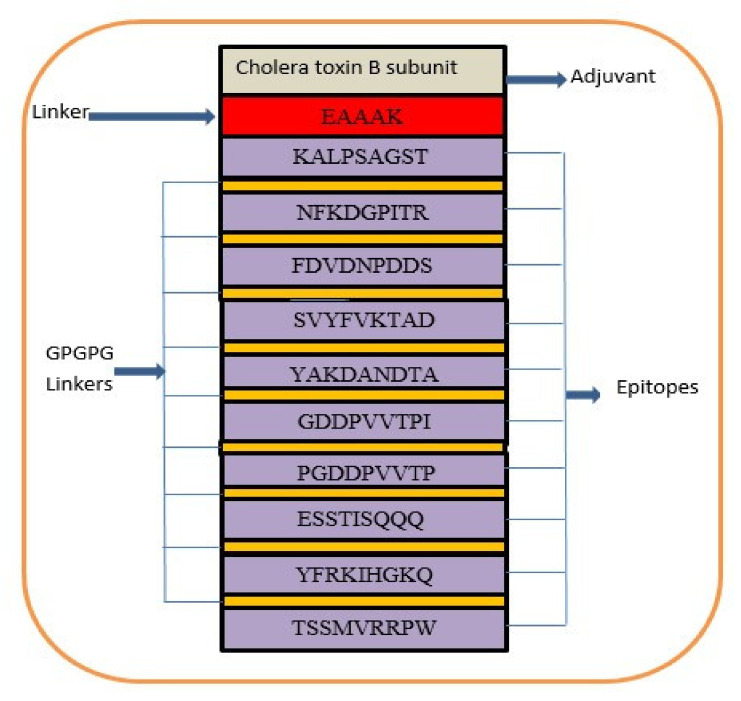
Schematic presentation of final vaccine epitope construct.

A multi-epitope-based vaccine construct consists of different types of epitopes, rather than a single epitope. The vaccine construct is designed by linking screened epitopes with each other through specific linkers, i.e., GPGPG linkers for the purpose of overcoming the limitations of single-peptide-based vaccines that are unable to generate effective immune responses against variants of the same pathogen [97]. Another linker, i.e., EAAAK, is used to link the adjuvant CTBS, to enhance the immune efficacy of the vaccine construct [98]. These specific linkers are used because they are rigid and allow for the separation of epitopes that have efficiently been recognized by the immune system [99]. Consequently, safe, robust, and efficient immune responses are generated against the designed vaccine [64]. The designed multi-epitope vaccine construct is mentioned in Figure 4. The amino acid length of the designed vaccine is 264. 

### 3.14. Structure Modeling

**Figure 5:**
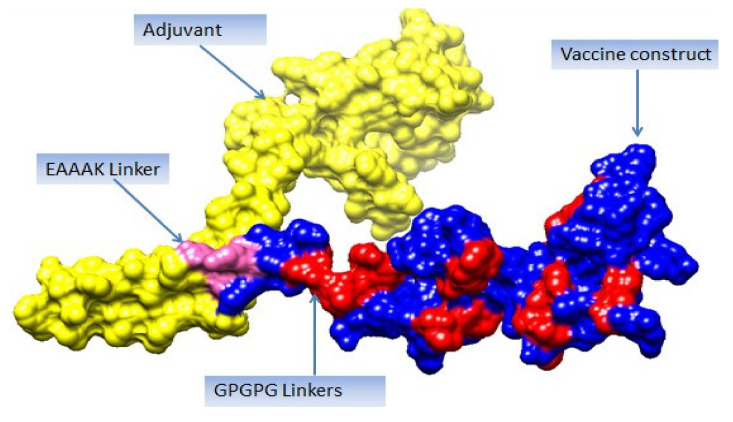
Vaccine 3D structure. Yellow color represents adjuvant (cholera toxin B subunit), blue color shows vaccine construct, and pink color represents EAAAK linker, while red color represents GPGPG linkers.

The three-dimensional structure of the vaccine was modeled using 3Dpro [100], as shown in Figure 5. The structure modeling was performed ab initio rather than by homology-based or threading because no appropriate template structure was available. 

### 3.15. Loops Modeling and Refinement 

Structure stability is necessary for a good vaccine candidate [101]. To avoid structure instability, all the present loops in the vaccine candidate were modeled for the following residues: Met1-Leu4, Ala19-Gly21, Csy30-Ile38, Ser51-Asn65, Gly66-Val73, Glu100-Asn111, Leu132-Thr151, Arg152-Gly171, Ser172-Asn191, Asp192-Gly211, Pro212-Pro226 and Ser230-Gln235, Gln236-Gly255, and Thr256-Trp264.

### 3.16. Disulfide Engineering

**Figure 6:**
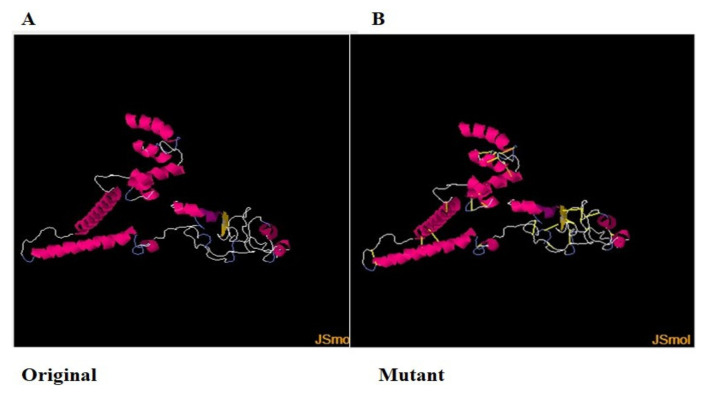
(**A**). Original wild structure of the vaccine construct and (**B**) mutated structure of the vaccine. The yellow sticks are the disulfide bonds introduced via disulfide engineering.

For structure stability, disulfide engineering was performed [102]. The covalent bonds ensure protein structure stability, and therefore, the geometry of the construct remains intact [103]. Moreover, some amino acid residues are sensitive to enzyme degradation. Hence, all the enzyme-degradable amino acid residues were replaced with cysteine residues, which are shown as yellow sticks in Figure 6B [104].

### 3.17. Codon Optimization

**Figure 7:**
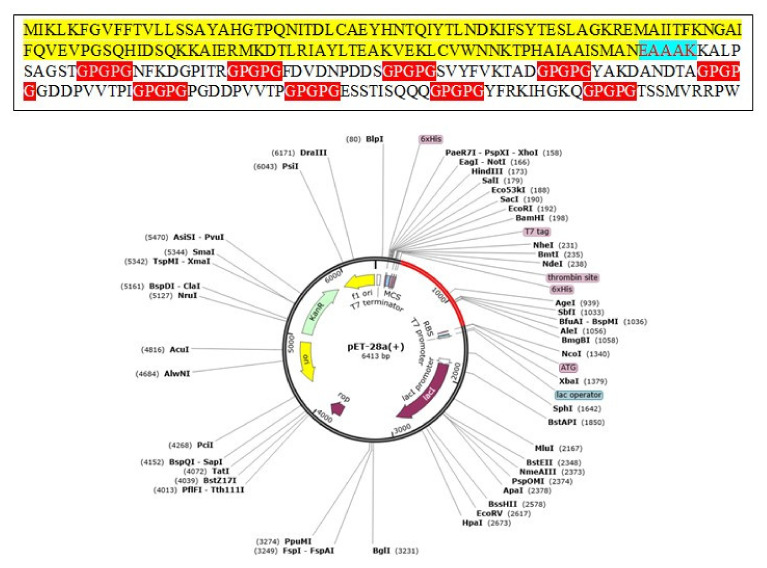
Cloning of multi-epitope vaccine constructs computationally into pET28a (+) vector. The vaccine is shown in red color.

Codon optimization is a specific genetic engineering technique that makes sure the codon optimization of the construct is consistent with the host immune usage pattern for maximum production and expression of proteins. The codon adaptation index threshold value is 0.92 and GC content is 57.08%. The values of CAI are estimated to be ideal for maximum expression and production of proteins [105,106,107]. Finally, pET-28a (+) expression vector was used to optimize the expression of the vaccine. The in silico cloned vaccine construct is shown in Figure 7.

### 3.18. Molecular Docking

Interaction of the designed vaccine construct with both innate and adaptive immune cells of the host is imperative for activation of efficient cellular and humoral immunity. Therefore, a docking study was performed to predict the binding affinity of the vaccine with host immune receptors [108]. A blind docking approach was carried out to evaluate the binding affinities with MHC-I (PDB ID:1L1Y) and MHC-II (1KG0) and TLR-4 (PDB:4G8A), which are receptors of the host [109]. Results were obtained from PatchDock server in which 20 resultant solutions were produced, as tabulated in Tables S3–S5 [110].

### 3.19. Refinement of Docked Complexes 

**Figure 8:**
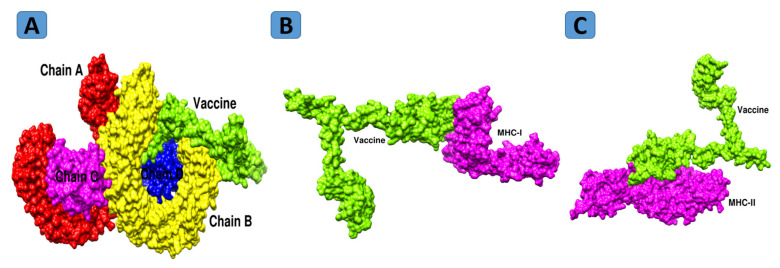
Docked conformation of vaccine with TLR4 (**A**), MHC-I (**B**), and MHC-II (**C**).

Results of PatchDock were additionally subjected to refinement. The complexes with the lowermost global energy were ranked top and selected further for binding mode and interaction studies through UCSF Chimera 1.13.1 [111]. For each receptor, the top-docked solution was selected. In the case of MHC-I, solution 8 was selected, as it has the lowest global energy of −12.72 kJ.mol^−1^ with good contribution from attractive van der Waals (−6.67 kJ.mol^−1^), repulsive van der Waals (0.73 kJ.mol^−1^), ACE (1.39 kJ.mol^−1^) and hydrogen bond (−0.43 kJ.mol^−1^) energy. Similarly, for MHC-II and TLR4, solutions 2 and 5 were selected based on the above criteria. [112]. The FireDock refinement results for MHC-I, MHC-II and TLR4 are shown in Tables S6–S8, respectively. The docked intermolecular conformation of the vaccine with MHC-I, MHC-II, and TLR4 is shown in Figure 8.

### 3.20. Chemical Interactions of Vaccine to Immune Cells Receptors 

Interaction between vaccine and host immune cell receptors is crucial in order to generate proper immune responses [113]. The chemical interactions between vaccine construct and TLR-4, MHC-I and MHC-II immune receptors were determined using the protein–peptide molecular docking approach [114], and specific residue-wise interactions of MHC-I, MHC-II and TLR 4 were checked using the UCSF chimera tool [111]. The model vaccine construct showed interactions with different residues of MHC-I within 3 Å. These interactions are both hydrophobic and hydrophilic. The interactions are shown in Table 3. Similarly, the vaccine also produced a strong interaction network with the MHC-II molecule. All the interactions are within close distance and are of different types, including hydrogen bonding, salt-bridges and van der Waals interactions [115]. The interacting residue network of the vaccine to MHC-II is presented in Table 3. Toll-like receptors are a class of several proteins that initiate acquired and adaptive immune response; among them, TLR-4 is one of the members of the TLR family, usually expressed on dendritic and macrophage cells [116]. The interacting residues of the model vaccine to TLR-4 are mentioned in Table 3.

### 3.21. Molecular Dynamic Simulation

**Figure 9:**
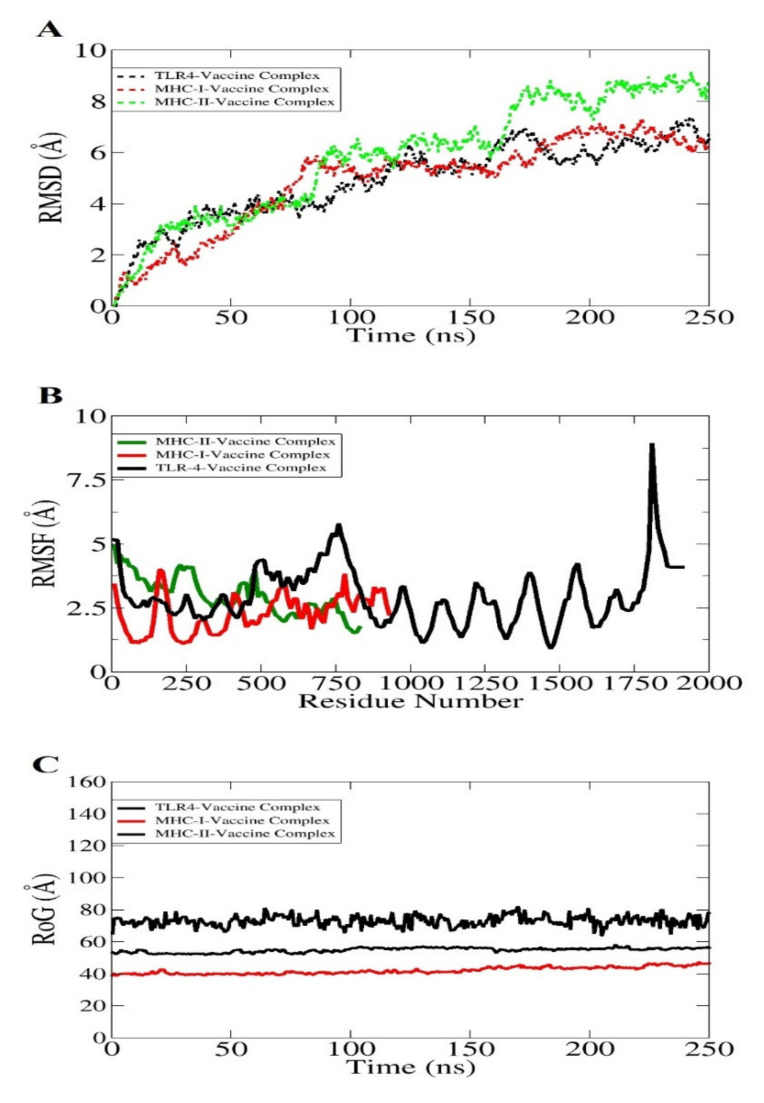
Different statistical analyses of the simulation trajectories. RMSD (**A**), RMSF (**B**), and RoG (**C**).

The molecular dynamics simulation is an in silico simulation for analyzing the dynamic behavior of macromolecules. In this system, the atoms and molecules are allowed to interact for a specific time period, giving a view of the dynamic “evolution” of the system. In the most common version, the trajectories of atoms and molecules are determined through numerically solving Newton’s equations of motion [117]. The docked complexes were analyzed in 250 nanosecond periods, whereas it is an important step to obtain and to ensure the binding affinity of the vaccine construct with dock receptors, i.e., MHC-I, MHC-II, and TLR-4 in a specific time [118]. However, it is mandatory to ensure that the vaccine antigens are efficiently exposed and recognizable by the host immune system to develop a robust immune response. No drastic changes were observed throughout the simulation period, as shown in Figure 9. The first analysis performed was root mean square deviation (RMSD) based on carbon alpha atoms. All three systems revealed increasing RMSD. The TLR4 and MHC-I system with the vaccine showed good binding stability compared to the MHC-II vaccine complex (Figure 9A). The mean TLR4-vaccine and MHC-I-vaccine RMSD values are ~4 and 4.2 angstrom, respectively. The MHC-II–vaccine complex reported a mean RMSD of 5.8 angstrom. The deviation in the systems is because of a larger size and a high number of flexible loops. This is evident in the root mean square fluctuation (RMSF) analysis (Figure 9B). The system’s intermolecular stability can also be witnessed by the radius of gyration (RoG), which reflected the highly compact nature of the vaccine–immune receptor complexes (Figure 9C).

### 3.22. Hydrogen Bonding

**Figure 10:**
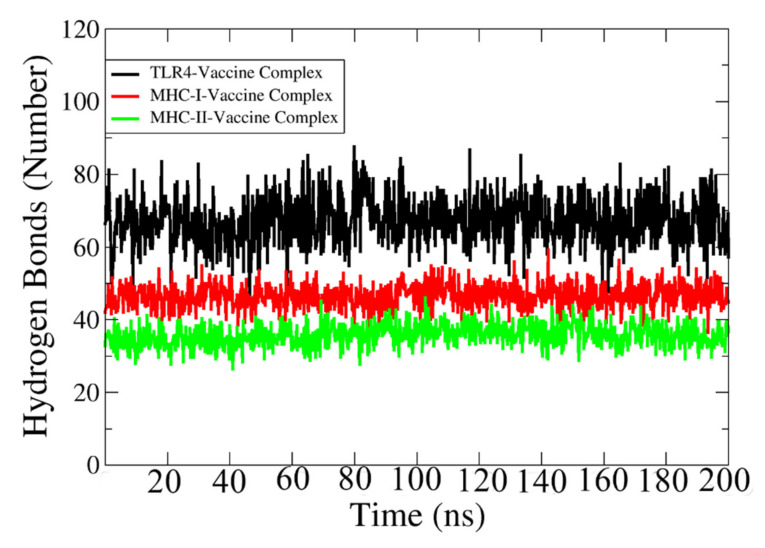
Number of hydrogen bonds between TLR-4, MHC-I, and MHC-II with designed vaccine construct.

Hydrogen bonds are formed between electronegative charged atoms of hydrogen and other electronegative charged particle atoms. Hydrogen bonds are non-covalent forces among electronegative atoms [119]. These bonds are formed between electronegative acceptors and donors. The VMD plugin was used to identify and count the hydrogen bonds between the vaccine and receptors formed in the simulation process [120], which is shown in Figure 10. The cut-off distance value is 3 Å. In each case, a strong and high number of hydrogen bonds (>35) are revealed between the vaccine and its relevant immune receptor.

### 3.23. Binding Free Energies Calculation

The MM-GB/PBSA approach was used to calculate dock complexes binding free energies [73]. The total free binding affinities for the receptor TLR4 with vaccine construct was −90.9 kcal/mol, while with MHC-I and the vaccine construct was −75.07.44 kcal/mol and the MHC-II with its vaccine construct was estimated to be −77.82 kcal/mol. In the MM-GB/PBSA, the net electrostatic and van der Waal energies are the most favorable in the complex formation. The overall gas phase energy dominates all three complexes. On the other side, polar energy is non-favorable and is non-polar in the complex formation. The different binding energy terms are tabulated in Table 4. 

### 3.24. In Silico Immune Simulation

For in silico immune simulation, the C-ImmSim server was used to evaluate the immune protective potency of the designed vaccine construct stimulating the host immune system using in silico approaches [121]. The position-specific score matrix (PSSM) is used by this approach, along with the other machining learning techniques to study and prioritize epitopes and their immune interactions [122]. In this analysis, the maximum level of exposure to the vaccine antigen was made certain for about 350 days regarding the human immune system. Hence, there was an enhancement in the provocation of adaptive immunity, which can be detected by high IgG and IgM antibody production. The IgM antibody level was also detected to be high. Secondary immune responses subsequently followed by tertiary immune responses led to the maximum production of B-cells and the high levels of IgM + IgG, IgM, IgG1 + IgG2, and IgG1 and IgG2, as shown in Figure S2A. Similarly, the production of interferon–gamma that was greater than 400,000 counts per ml for almost 350 days was observed, as shown in Figure S2B. The humoral and cellular immune responses to the vaccine are presented in Figure S3. Similarly, the response of different immune cells to the vaccine is shown in Figure S4.

## 4. Discussion

The multi-drug resistant microbes are evolving rapidly and are causing fatal infections not only in humans but in other organisms as well. When bacteria evolve, the efficacy of the drugs is reduced, and hence, AMR develops [123]. Vaccination is considered one of the most important alternatives and effective methods for the prevention of bacterial infections. It is of dire need to develop alternative methods to counter multi-drug resistant bacteria because the presently available antibiotics have become inefficient [124]. In the past decade, almost half of the antibiotics have become ineffective against the present evolving pathogens. Experimental vaccination is time consuming and requires a longer period. Conversely, computational methods along with the advancement in genomic sciences has brought many-fold progress in developing broad spectrum vaccines against AMR pathogens in considerably less time and using less resources [125]. 

*P. rettgeri* is resistant to many antibiotics such as ampicillin, polymyxin, first-generation cephalosporins, gentamicin, tobramycin, carbapenem, and tigecycline [36,37,38]. Additionally, it is susceptible to amikacin, according to one study [34]. Furthermore, metallo-β-lactamase-1 and amoxicillin-clavulanate resistivity patterns are noted [40]. The MDR *P. rettgeri* genome sequence revealed that the pathogen harbors 17 resistance genes and various virulence and heavy metal resistance genes [126]. The high resistance spectrum of *P. rettgeri* has prompted research to use genomic information of the pathogen to devise new therapeutic strategies, in particular, a vaccine to stop the spread of MDR strains and effectively manage its infections [127]. 

Vaccine preparation is effective and is the most successful method to prevent infection in a host against pathogenic microbes [128]. The bioinformatics approach is an easier method to design an in silico vaccine. The step-wise rationale of a computational vaccine design approach consists of the following steps: (i) target antigen identification, (ii) select vaccine platform, (iii) optimize gene expression factors, (IV) construct a vaccine candidate, and (v) check immunogenicity and efficacy of vaccine construct and ensure its biological safety, which must be evaluated by using cellular models in vitro and animal models [129].

Traditional and conventional vaccine development is based on Pasteur principles that consist of isolation, inactivation, and injection of non-virulent pathogenic microbes or part of the microbe into the host [130]. Conventional vaccines are vital pharmacological products, but production and development are expensive, not only economically; they also require much time. It takes years to prioritize a potential vaccine candidate against particular infectious microbes [131]. Particularly, the traditional vaccine development phase requires time and grants for making good antigenic candidates. Besides, there are chances of adverse autoimmune side effects. Moreover, the culturing of pathogenic fatal microbes is risky to deal with in laboratory conditions. Therefore, to reduce cost and time, along with eliminating the risk of spreading infections, vaccinomic applications and tools have been established for the development of a vaccine [132]. Potential vaccine candidates are rapidly developed with the aid of computer-aided software/prescreening servers. Various types of vaccines have been designed through in silico approaches, i.e., COVID-19, Dengue, Cancer, *Salmonella typhi*, and Meningitis [133,134].

The current work is based on a multi-epitope vaccine against *P. rettgeri*. The potential surface membrane and secretory proteins were prioritized using bioinformatics tools. The complete proteome of *P. rettgeri* was retrieved from the NCBI databases. BPGA tool core genes from the genomic pool of *P. rettgeri* were extracted, followed by various types of analyses to prioritize potential vaccine candidates: (1) CD-hit analysis, (2) homology check, (3) essential check, and (4) localization check. Furthermore, the virulence factors were checked via VFDB, followed by transmembrane helices checked with HMMTOP 2.0 and TMHMM 2.0 web servers. Blastp server was used to check for homology with the host genes and normal flora of the host. The physicochemical properties calculated were computationally evaluated with the ProtParam server and ExPasy tool. Vaxign 2.0 webserver was used to check adhesion probability. An antigenicity check was performed to prioritize highly potential vaccine candidates with the aid of Vaxijen 2.0. Moreover, allergenic sequences were found through Allertop 2.0 web server and excluded from the study. B-cell and B-cell-derived T-cell epitopes were predicted from shortlisted filtered proteins. All the predicted epitopes were linked with each other to design multi-epitope vaccine, as highlighted by Ismail et al., 2020. Multi-epitope-based vaccines can combat a wide range of infectious caused by *P. rettgeri* strains. Multi-epitope vaccines are consistent of several different types of epitopes versus single epitopes, and they have the capacity to generate both humoral and cellular immunity. The designed multi-epitope vaccine was docked with different immune cell receptors. The interaction of the vaccine with immune cells is important in generating a proper immune response; hence, these interactions were analyzed through molecular docking. The docking results were validated through molecular dynamics simulation and binding free energies calculations. Results of the molecular dynamic simulation revealed no drastic changes throughout the simulation time, which is important for the recognition of peptides by the immune system in order to provoke an immune response. Overall, this study concluded that the designed vaccine construct can tackle infectious caused by *P. rettgeri*. However, in vivo and in vitro experiments will further support the outcomes of this study. 

## 5. Conclusions and Limitations

The excessive use of antibiotics in humans and animal medicine, agriculture, and the environment has led to AR in bacteria, which has significantly contributed to high hospital and community mortality and mobility. AR in *P. rettgeri* leas to life-threatening health issues around the globe and is becoming difficult to treat. To tackle infections of the pathogen, no significant work of vaccinology is under process. The use of bioinformatics webservers will not only lessen the cost of vaccine research work but will also reduce the time to identify vaccine targets. In this study, a pan-secretome and pan-exoproteome-based recombinant vaccine candidate was designed using immunoinformatics and reverse vaccinology approaches that will induce robust immune responses against *P. rettgeri*. For the above purpose, potential antigenic and highly non-allergenic surface membrane peptides epitopes were selected. With the aid of various immunoinformatics web tools, it is suggested that this vaccine candidate might provoke strong and active immunologic immune responses in the host. Furthermore, wet laboratory experimental applications are recommended to validate these in silico predictions. It is hoped that this research might help to develop the interest of scientists and researchers in the bioinformatics field on the above subject. Despite the promising results of the study, several methodological limitations can be overcome in the future. For example, the use of more refined tools/servers in terms of algorithms to validate the predictions. Similarly, the optimal ordering of epitopes in the vaccine construct needs strong experimental proof. Lastly, experimental validation of in vivo and in vitro vaccine models is a must. 

## Figures and Tables

**Table 1:** Physiochemical properties of shortlisted proteins. Molecular weight (MW), isoelectric point (PI).

Vaccine Target	Physiochemical Properties					
Extracellular Proteins	Amino Acid	GRAVY	Aliphatic Index	Instability Index	PI	MW
>core/4909/2/Org2_Gene2897 (fimbrial protein)	176	0.152	94.37	15.92	5.03	18.07
>core/1455/14/Org14_Gene1001(flagellar hook protein FlgE)	422	−0.405	68.41	20.52	4.53	44.53
Outer Membrane Proteins						
>core/3432/1/Org1_Gene4222 (flagellar basal body L-ring protein FlgH	260	−0.229	85.54	37.68	9.21	27.93
Periplasmic Proteins						
>core/6354/1/Org1_Gene719 (flagellar hook-basal body complex protein FliE)	114	−0.179	91.4	40.73	5.29	12.72
>core/4058/2/Org2_Gene3304 (flagellar basal body P-ring formation protein FlgA)	234	−0.259	89.57	34.84	9.68	26.13
>core/3402/4/Org4_Gene1551(Gram-negative pili assembly chaperone domain proteins	273	−0.478	79.3	35.34	9.45	30.48

**Table 2:** List of probable antigenic, good water-soluble, non-toxigenic, and non-allergic DRB*0101 binding affinity epitopes.

Selected Epitopes	DRB*0101 IC_50_ Predicted Score	Antigenicity	Solubility	ToxinPred	Allergenicity
KALPSAGST	20.94	0.6431	Soluble	Non-toxic	Non-allergen
NFKDGPITR	88.92	0.9418			
FDVDNPDDS	4.2	0.5464			
SVYFVKTAD	94.19	0.6127			
YAKDANDTA	22.13	0.9316			
GDDPVVTPI	35.97	0.8934			
PGDDPVVTP	52.48	0.8771			
ESSTISQQQ	22.8	1.0044			
YFRKIHGKQ	21.33	1.3281			
TSSMVRRPW	20.45	0.695			

**Table 3:** Residue-wise interactions of vaccine to MHC-I, MHC-II, and TLR-4.

Vaccine Complex	Interactive Residues
MHC-I	GLU128, ARG111, ARG65, LYS68, HIS168, GLU4, LYS3, GLN155, HIS67, GLY8, TYR80, YR116, ALA117, ARG144, TYR1, ALA69, VAL67, LYS68, GLU19, VAL76, GLN72, PRO5TYR73, VAL6, TYR159, TRP147, LYS146, ALA150, TYRE84.
MHC-II	SER77, ARG66, TYR32, ASN33, ILU82, ASN84, THR83, THR133, PRO86, PRO87, PHE145, LEU138, ASP57, PRO61, PHE109, ARG72, SER110, PHE116, TYR107, GLE113, CYS115, ARG220, TYR104, TYR101, THR100, CYS138, PRO137, TYR139, TYR136, SER42, ASP41, VAL44, ARG25, GLU141, HIS16, ASP116, HIS143, GLN36, ARG40, LEU144, LYS111, PRO86, ASN62, TYR160
TLR-4	GLN599, ARG589, GLU60, GLY617, GLU485, PRO88, LEU87, ILE80, LYS91, LEU78, VAL135, ASP70, LYS84, GLU87, ARG88, THR92, ALA131, LYS130, SER134, PRO140, GLY143, PHE145, ASP147, ILE150, ALA97, ASP189, ALA187, ALA190, THR193, GLY181, GLY171, ASN65, VAL33, GLU31, LEU37, ILE80, LEU78, LYS91, GLU143, LYS89, ASN86, PHE151, GLU48, ALA462, PRO88, TYR540, ASN526, GLN37, THR11, GLN597, ASP596, VAL604, GLN37, VAL8, GLU 03, ASP29, PHE10, ASP580, GLN547

**Table 4:** MMGBSA/PBSA binding free energies results from the vaccine construct with MHC- I, MHC-II, and TLR4 complexes. The energy values are reported in kcal/mol.

Energy Parameter	TLR-4–Vaccine Complex	MHC-I–Vaccine Complex	MHC-II–Vaccine Complex
MM-GBSA			
VDWAALS	−78.13	−72.88	−70.18
EEL	−69.74	−51.12	−60.57
EGB	65.10	57.08	60.11
ESURF	−8.13	−8.15	−7.18
Delta G gas	−147.87	−124	−130.75
Delta G solv	56.97	48.93	52.93
Delta Total	−90.9	−75.07	−77.82
MM-PBSA			
VDWAALS	−78.13	−72.88	−80.24
EEL	−69.74	−51.12	−62.58
EPB	69.10	48.97	56.67
ENPOLAR	−6.40	−7.10	−9.18
Delta G gas	−147.87	−124	−130.75
Delta G solv	62.7	41.87	47.49
Delta Total	−85.17	−82.13	−83.26

## Data Availability

The data presented in this study are available within the article.

## Supplementary Materials

The following supporting information can be downloaded at: https://www.mdpi.com/article/10.3390/vaccines10020189/s1, Figure S1. (A) Core pan plot of 14 *P. rettgeri* genomes. (B) Pan-phylogeny tree of 14 complete genomes of *P. rettgeri*. Figure S2. (A) Antibody titer as shown in different color peaks in response to the vaccine injection (black color peak). (B) Simulation of interleukins and interferon level after injection of vaccine. Figure S3. Different B- and T-cell responses against the vaccine. Figure S4. Different immune cell responses are generated in response to the chimeric vaccine construct. Tc (cytotoxic killer T-cell), macrophages (Mφ), natural killer cells, dendritic and epithelial cells. Table S1. Predicted B-cell epitopes. Table S2. MHC-I and MHC-II predicted epitopes with a percentile score. Table S3. Docking score of top 20 docked vaccine complex with MHC-I molecule. Table S4. Docking score of top 20 docked vaccine complex to MHC-II molecule. Table S5. Docking score of top 20 docked vaccine complex to TLR4 molecule. Table S6. Top 10 refined docked complexes of vaccine–MHC-1 complexes. Table S7. Top 10 refined docked complexes of vaccine–MHC-II. Table S8. Top 10 refined docked complexes of vaccine-TLR4.
