# Supplementary material for: Designing a Recombinant Vaccine against Providencia rettgeri Using Immunoinformatics Approach
Source: Vaccines (Basel). 2022 Jan 25;10(2):189. doi: 10.3390/vaccines10020189 (PMC8876559; doi:10.3390/vaccines10020189)
Supplement: Supplementary file 1 [file vaccines-10-00189-s001.zip › vaccines-1560462-supplementary.pdf]

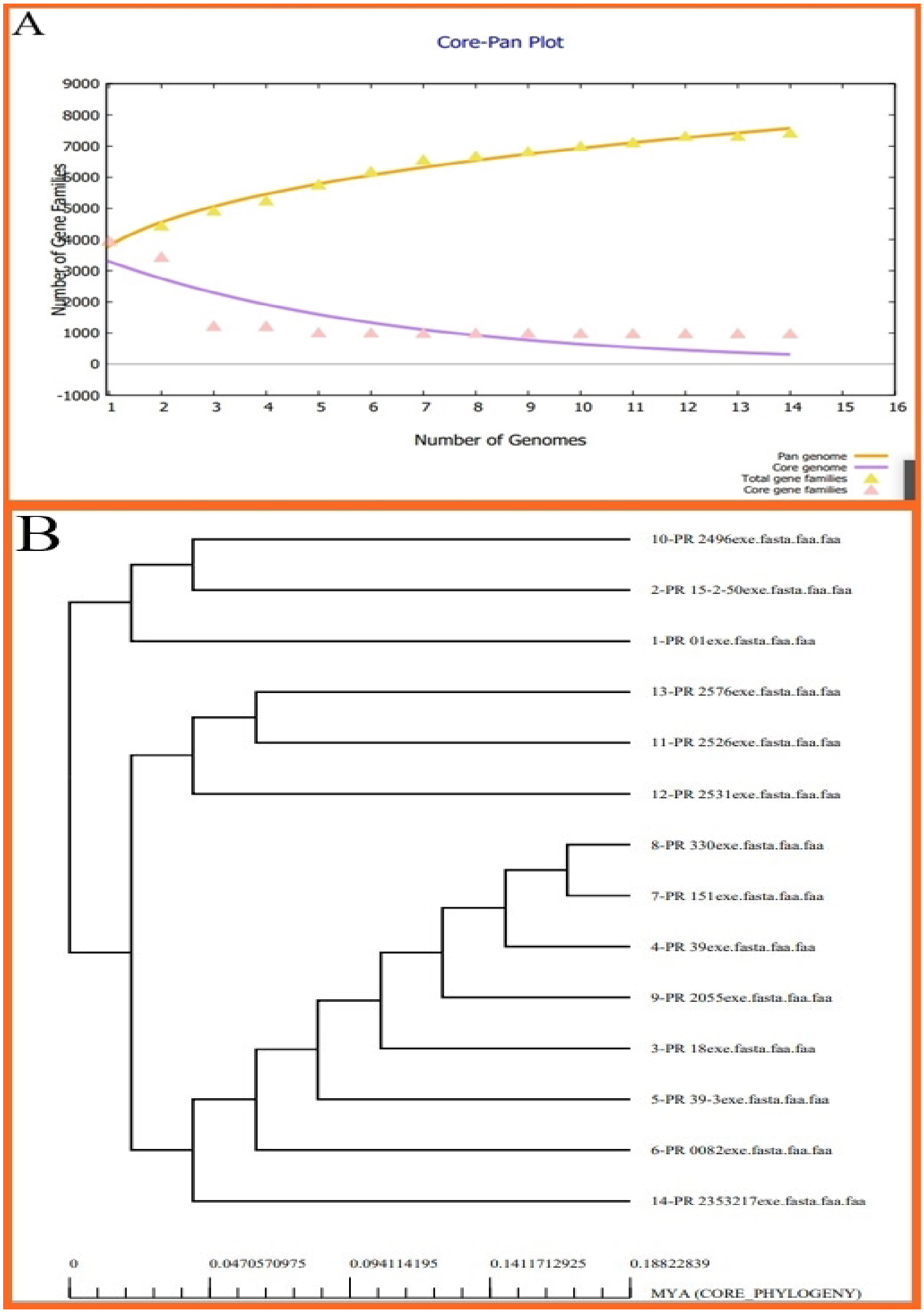

Figure S1. (**A**) Core pan plot of 14 *P.rettgeri* genome. (**B**) Pan-phylogeny tree of 14 complete genomes of *P.rettgeri*.

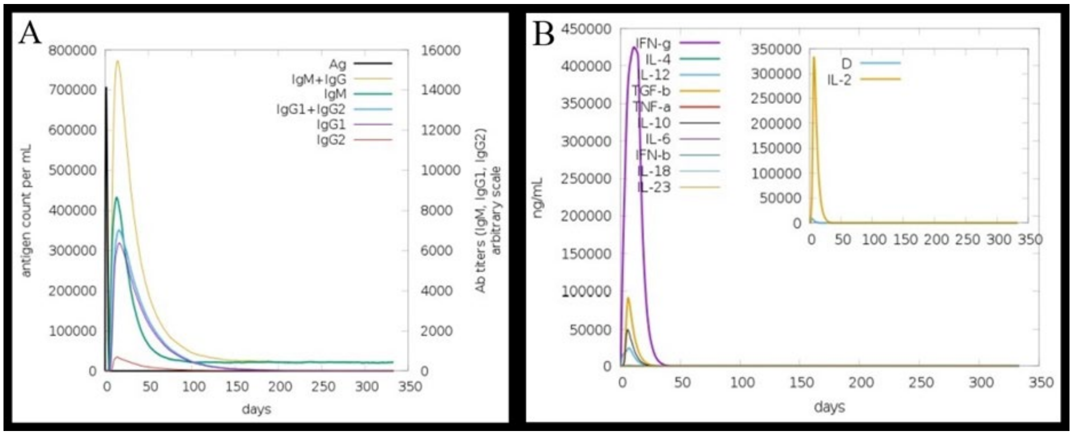

Figure S2. A. Antibody titer as shown in different color peaks in response to the vaccine injection (black color peak)**. B** Simulation of interleukins and interferon level after injection of vaccine.

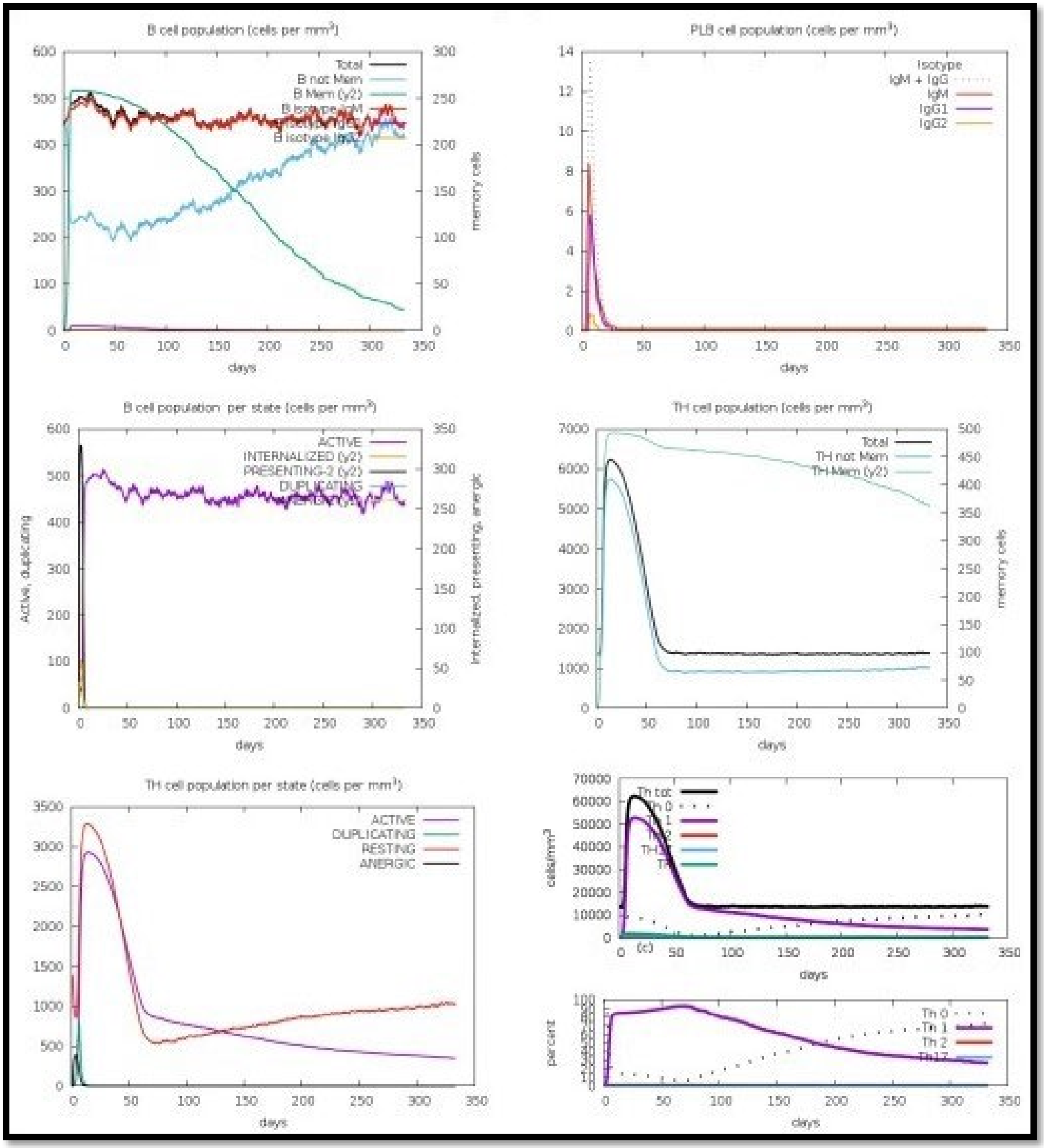

Figure S3. Different B and T-cell responses against the vaccine

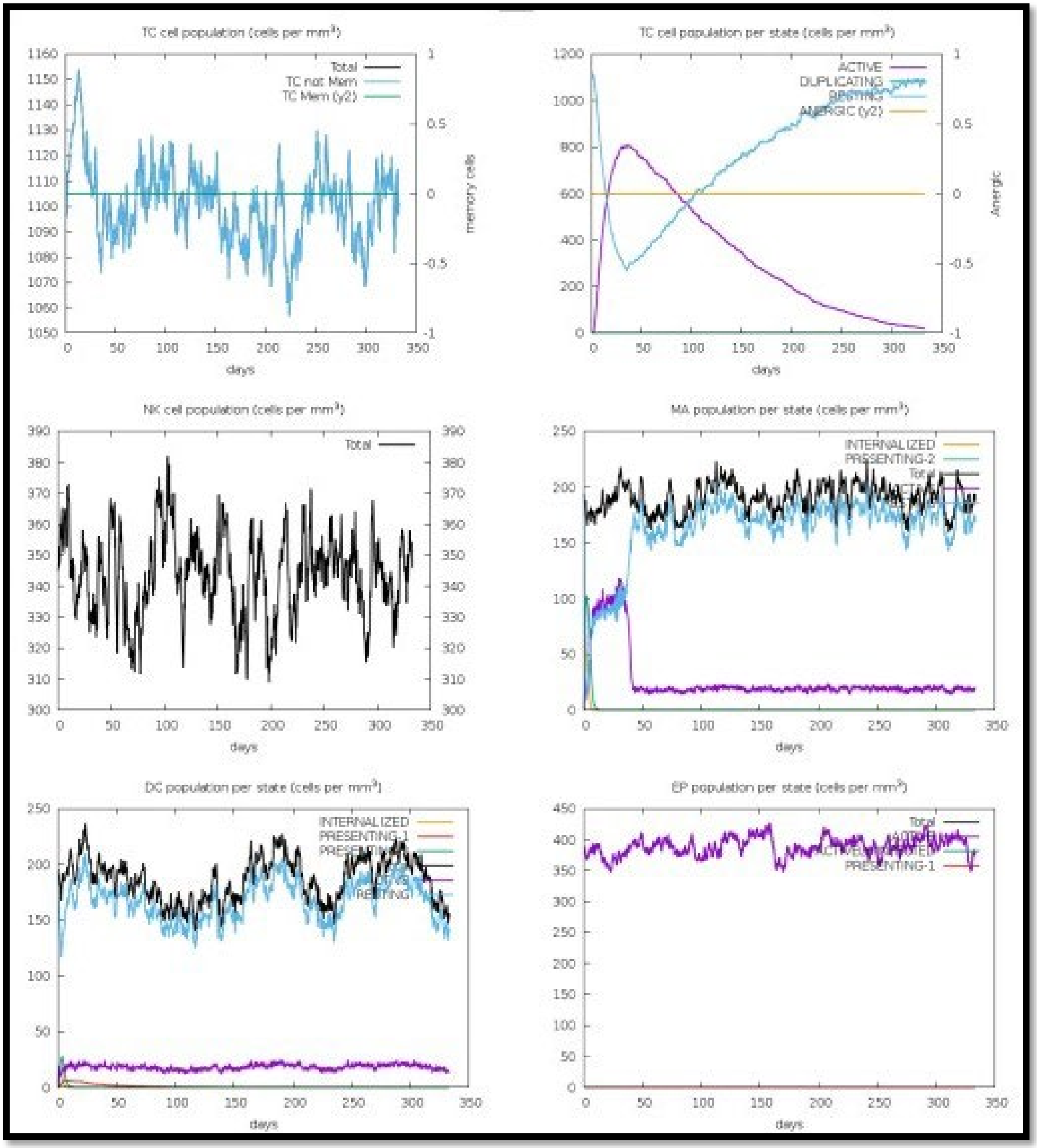

Figure S4. Different immune cell responses are generated in response to the chimeric vaccine construct. T_c (cytotoxic killer T-cell), macrophages (Mφ), natural killer cell, dendritic and epithelial cell.

Table S1. Predicted B-cell epitopes**.**

**Protein**	**Predicted B–cell epitopes**
>core/4909/2/Org2_Gene2897 Fimbrial protein	LASGSDALKV
	LGKVSKKALPSAGSTAAA
	DATSYPGDDSVIELKDAGQSGVA
	VIPLFTASKAYPLKDGVENN
	IAQTANVGV
>core/1455/14/(Org14_Gene1001 Flagellar hook protein FlgE)	TNGFKGATTSFADMFAGSGVGLG
	TQNFKDGPITRTD
	YPASVDEKGNVTIQSGGVPEGLNIPTDMMDAKAS
	INLNSEDKIKDKAFDVDNPDDSATYNFSTTMTAYDSQGNTHEISVYFVKTADNKWTAYAKDANDTAATKLGDMSFDGNGKLVTETVTPGPNPGDDPVVTPIETTFDFAYKGLNGAND
	LGKTRQQKVSESSVSAIDVNGYPAGEYTTF
	PNGLVSQGGNVWASSNASGNPMDGVPGVGQFGK
>core/3432/1/Org1_Gene4222 (Flagellar basal body L-ring protein FlgH)	TLQQKSSMMIESSTISQQQKIALQTPLI
	HIKSRPLVDTQTTAIPSAPTAPAPNGSIFQSAQPAYYGYQPLFEDRRPRNVG
	VSASKNSSANANRTGKTGFLAAILPGFMQGWIGGKNTELDIKGNSDFSGKGGANAN
	DGYINESQNMGW
>core/6354/1/Org1_Gene719 (Flagellar	SQLNVVATQATNPTKPIIEPVG

hook-basal body complex protein FliE)	NDSAKQVENFTLGKQDISL
>core/4058/2/Org2_Gene3304 (Flagellar basal body P-ring formation protein FlgA)	PQDIDQYFRKIHGKQNSV
	IEIKTPIDKWPNCDKPQIGLPSGGRNM
	NIQKGETIEFVDIGTKKGLIHQLPSGASTDKMALR
	LRNITAGQTFTSSMVRRPWA
	INNAAMGEN
>core/3402/4/Org4_Gene1551 (Gram-negative pili assembly chaperone domain protein)	MMKNKLMNKKNAH
	SIIPTREQMDNPWQ
	PNKNGQTVAGFE

Table S2. MHC-I and MHC-II predicted epitopes with a percentile score

**MHC I epitopes**	**Percentile score**	**MHC II epitope**	**Percentile score**
LPSAGSTAA	0.29	KALPSAGSTAAA	2.4
KALPSAGSTA	2.6		
GDDSVIELK	3.4	PGDDSVIELKDA	15
DSVIELKDA	9		
FTASKAYPL	0.09	PLFTASKAYPL	0.01
PLFTASKAY	0.33		
GFKGATTSF	0.24	NGFKGATTSFA	0.16
NGFKGATTSF	0.77		
MFAGSGVGL	1.1	DMFAGSGVGLG	1.4
DMFAGSGVGL	1.9		
NFKDGPITR	0.01	TQNFKDGPITRTD	28.07
TQNFKDGPI	6.8		
VDEKGNVTI	2.1	VDEKGNVTIQSG	3.4
KGNVTIQSG	9.9		
GLNIPTDMM	1.9	GVPEGLNIPTDMM	27
GVPEGLNIPT	4.8		
ATYNFSTTM	0.06	ATYNFSTTMTAYDSQ	2
TTMTAYDSQ	4.3		

KIKDKAFDV	0.39	KIKDKAFDVDNPDDS	5.5
FDVDNPDDS	26		
NTHEISVYF	0.03	GNTHEISVYFVKTAD	12
SVYFVKTAD	10		
YAKDANDTA	1.5	NKWTAYAKDANDTAA	6.8
NKWTAYAKDA	44		
ATKLGDMSF	0.12	ATKLGDMSFDGNGKL	5.8
MSFDGNGKL	0.23		
ETVTPGPNP	1.1	KLVTETVTPGPNPGD	14
KLVTETVTP	2.3		
VVTPIETTF	0.05	PGDDPVVTPIETTFD	20
PGDDPVVTPI	13		
KVSESSVSA	0.46	QKVSESSVSAI	5.4
KVSESSVSAI	0.83		
YPAGEYTTF	0.01	DVNGYPAGEYTTF	13
DVNGYPAGEY	0.04		
LVSQGGNVW	0.09	LVSQGGNVWAS	2.2
SQGGNVWAS	2		
NPMDGVPGV	0.08	GNPMDGVPGVGQFGK	14
GVPGVGQFGK	0.43		

Table S3. Docking score of top 20 docked vaccine complex with MHC-I molecule.

**Solution No**	**Score**	**Area**	**Atomic Contact Energy (Kj/mol)**
1	19720	2869.6	340.01
2	18564	2649.9	152.45
3	18370	2385.9	486.55
4	18312	3332.6	-110.49
5	18134	2767.6	366.92
6	18122	3006	240.11
7	18114	2655.7	419.53
8	17984	3034	207.03
9	17944	2750.6	484.53
10	17828	2353.8	329.07
11	17680	2694.6	497.52
12	17348	3205.9	115.70
13	17334	3081.2	100.24
14	17308	3378.6	387.97
15	17216	2519.6	343.27
16	17138	4053.4	43.63
17	17056	2934.2	88.51
18	16882	3289.2	204.41

19	16804	2521.1	439.43
20	16752	2161.9	397.81

Table S4. Docking score of top 20 docked vaccine complex to MHC-II molecule.

**Solution No**	**Score**	**Area**	**Atomic Contact Energy (Kj/mol)**
1	18648	2946.4	206.29
2	17954	2914.1	213.33
3	17572	2758.3	204.33
4	17488	2844.3	290.31
5	17434	2296.2	137.21
6	17230	2552.9	174.45
7	17148	2242.9	376.22
8	17130	2984.2	287.60
9	17122	2585	-25.83
10	17078	3184.6	220.48
11	16968	2203.2	381.82
12	16946	3443.8	225.29
13	16636	2620.4	133.95
14	16606	3320.8	280.04
15	16486	2979.7	-35.23
16	16462	2336.2	-70.37

17	16368	2343.6	38.22
18	16258	2351.1	217.02
19	16158	2495.1	295.20
20	16078	2176.1	117.02

Table S5. Docking score of top 20 docked vaccine complex to TLR4 molecule.

**Solution No**	**Score**	**Area**	**Atomic Contact Energy (Kj/mol)**
1	21264	3863.6	231.13
2	20686	2786.4	321.49
3	19772	3454.7	95.62
4	18814	2775.7	438.07
5	18744	2886.8	454.84
6	18368	2859.5	409.96
7	18294	3199.8	320.28
8	18132	2452.3	499.25
9	18078	3140.3	-1.52
10	17648	3024.8	309.62
11	17580	2894.2	403.63
12	17478	2862.6	403.20
13	17110	2372.6	197.67

14	16942	3195.6	365.43
15	16940	2080.6	183.43
16	16908	2161.2	231.62
17	16858	2279.9	80.98
18	16782	3679.5	193.10
19	16748	2468.7	241.49
20	16692	2925.1	408.54

Table S6. Top 10 refined docked complexes of vaccine-MHC-1 complexes.

Rank	Solution Number	Global Energy	Attractive van der Waals Energy	Repulsive van der Waals Energy	Atomic Contact Energy	Hydrogen bond Energy
1	8	-12.72	-6.67	0.73	1.39	-0.43
2	4	1.96	-9.29	0.34	-0.80	-1.28
3	3	27.39	-4.55	6.23	6.96	-0.44
4	9	31.50	-25.67	12.89	10.98	-2.41
5	1	44.13	-26.56	64.95	14.15	-5.77
6	10	385.38	-32.47	468.30	9.96	-1.05
7	2	1105.21	-53.45	1431.20	12.34	-6.29
8	7	1135.23	-22.48	1412.47	8.00	-3.28
9	5	2746.08	-61.53	3514.66	25.26	-14.26
10	6	8950.81	-69.38	11339.83	-11.34	-4.87

Table S7. Top 10 refined docked complexes of vaccine-MHC-II.

Rank	Solution Number	Global Energy	Attractive van der Waals Energy	Repulsive van der Waals Energy	Atomic Contact Energy	Hydrogen bond Energy
1	2	36.23	-1.79	0.31	2.02	0.00
2	6	230.88	-11.98	258.67	7.34	-1.79
3	7	274.08	-15.30	350.15	12.35	-1.37
4	3	2061.33	-32.34	2553.77	22.85	-0.75
5	4	2122.83	-45.05	2708.40	4.16	-5.83
6	5	2177.67	-93.11	2926.27	-5.98	-13.45
7	9	3832.02	-43.55	4891.34	-4.23	-6.03
8	10	4044.56	-33.23	5060.52	11.17	-1.84

9	8	4426.42	-46.94	5572.97	6.57	-12.44
10	1	8857.37	-66.09	11187.25	7.04	-15.06

Table S8. Top 10 refined docked complexes of vaccine-TLR4.

Rank	Solution Number	Global Energy	Attractive van der Waals Energy	Repulsive van der Waals Energy	Atomic Contact Energy	Hydrogen bond Energy
1	5	6.97	-7.46	9.51	1.34	-0.85
2	8	8.93	-28.83	20.67	11.95	-3.48
3	6	35.64	-29.46	59.10	13.99	-4.69
4	2	88.80	-44.36	171.58	11.83	-3.82
5	4	167.70	-35.37	233.56	16.82	-1.66
6	10	291.89	-23.52	394.24	13.67	-3.94
7	3	355.79	-50.06	461.66	19.31	-6.29
8	9	612.36	-56.93	857.91	11.82	-5.28
9	7	776.79	-38.69	976.63	20.46	-5.25
10	1	5299.49	-64.47	6725.73	8.55	-9.14
